# Roles of SIRT3 in aging and aging-related diseases

**DOI:** 10.7150/ijbs.115518

**Published:** 2025-07-28

**Authors:** Yuzi You, Zhong Wang

**Affiliations:** 1 School of Clinical Medicine, Tsinghua University, Beijing 100084, China.; 2 Department of General Practice, Beijing Tsinghua Changgung Hospital, School of Clinical Medicine, Tsinghua University, Beijing 100084, China.

**Keywords:** SITR3, Mitochondria, Aging, Aging-related Diseases, Neurodegenerative Diseases

## Abstract

Aging is an inexorable pathophysiological progression characterized by the overwhelming deterioration of tissue integrity and cellular function coupled with increased risks of various aging-related diseases. Demographic shifts toward extended longevity have precipitated a paradigm shift in disease epidemiology, in which neurodegenerative conditions and cardiovascular pathologies now constitute predominant determinants of morbidity and mortality in geriatric populations. These conditions severely erode functional autonomy in aging populations and strain healthcare infrastructures globally.

As a principal nicotine adenine dinucleotide-dependent deacetylase within mitochondria, sirtuin 3 (SIRT3) exerts multimodal regulatory effects spanning mitochondrial bioenergetics, oxidative stress, and epigenetic modifications associated with aging. This review summarizes recent discoveries regarding the involvement of SIRT3 in physiological aging and its pathophysiological intersections with major aging-related disorders, providing new insights and ample inspiration for future research aimed at slowing the aging process and improving outcomes in aging-related diseases.

## Introduction

Sirtuins (SIRTs) comprise an evolutionarily conserved family of nicotine adenine dinucleotide (NAD^+^)-dependent deacylases that beneficially modulate lifespan and healthspan^1^. These enzymes orchestrate a wide range of physiological mechanisms that contribute to cellular and systemic homeostasis, encompassing DNA damage repair^2^, mitochondrial biogenesis^3^, genomic stability^4^, inflammatory response^5^, and metabolic homeostasis^6^. By integrating various stress response pathways, SIRTs serve as central regulators of cellular integrity and overall organismal well-being^7^. Consequently, these proteins have been identified as key determinants in the aging process and in the prevention of aging-related diseases.

Mammalian systems express seven phylogenetically conserved sirtuin variants (SIRT1-7), each occupying distinct subcellular compartments^8^, which underpins their diverse functional roles in cellular processes. Although these isoforms maintain a structurally conserved catalytic domain essential for enzymatic activity^9^, their divergent N- and C-terminal segments confer differential localizations and substrate specificities, enabling SIRTs to regulate a broad spectrum of cellular functions and respond to various physiological and environmental cues^10^. Originally characterized as class III histone deacetylases (HDACs), SIRTs are currently recognized as catalysts for multifaceted post-translational modifications, encompassing ADP-ribosylation, desuccinylation^11^, demalonylation^12^, depropionylation^13^, and debutyrylation^14^. These modifications allow SIRTs to exert profound epigenetic control over gene expression and regulate critical biological processes essential for cellular health^15^, such as maintaining genomic integrity^16^, controlling apoptosis^17^, metabolic adaptation^18^, managing inflammatory responses^19^, and combating oxidative stress^20^. Collectively, these activities have significant implications for the aging process and aging-related pathologies^21^.

Among SIRTs, SIRT3 is the only isoform directly linked to human longevity^22^. Its predominant localization in the mitochondria and its central role in energy metabolism make it a critical regulator of mitochondrial function and metabolic adaptation^23^. Human SIRT3 is encoded by a 399-residue polypeptide organized into two evolutionarily conserved functional domains, including an N-terminal Rossmann fold that binds NAD^+^ cofactors and a C-terminal zinc finger module essential for structural stabilization^24^. This unique structural configuration enables SIRT3 to catalyze several enzymatic reactions^25^, including deacetylation, ADP-ribosylasation, demalonylation, and desuccinylation^26^,^27^. Mitochondrial proteomic analyses have identified multiple substrates of SIRT3, confirming its pivotal role in regulating metabolic pathways, particularly under stress conditions such as fasting and exercise^28^.

Although the mitochondrial function of SIRT3 is well established, emerging evidence also indicates that SIRT3 translocates to the nucleus under certain conditions and helps maintain the heterochromatin structure and prevent cellular senescence, particularly in human mesenchymal stem cells (hMSCs)^29^. This dual subcellular localization positions SIRT3 as a sentinel regulator of mitochondrial-nuclear crosstalk, highlighting its versatility in maintaining cellular homeostasis across different compartments. SIRT3 also contributes to genomic stability and cellular longevity by facilitating reactive oxygen species (ROS) detoxification by activating superoxide dismutase 2 (SOD2)^30^ and promoting mitophagy^31^. These mechanisms substantiate SIRT3's pleiotropic functions in maintaining genomic stability and cellular longevity, reinforcing its therapeutic relevance in combating oxidative damage and mitigating the effects of aging.

This review aims to illuminate the structural and functional roles of SIRT3 in the aging process, particularly its involvement in major aging-related diseases. Furthermore, we explore the therapeutic potential of SIRT3, positioning it as a promising target for mitigating aging-related pathologies and highlighting its growing importance in the field of geroscience.

## Method

To ensure a comprehensive and systematic review, we conducted a literature search using databases such as Web of Science, Scopus, and Google Scholar for studies published up to June 2025. Keywords including “SIRT3,” “aging,” and “aging-related diseases” were used in various combinations. We primarily focused on peer-reviewed journal articles published in English. The inclusion criteria were relevance to the core themes of this review, publication within the last 10 years, and the inclusion of original research or substantial theoretical discussion. All cited references are provided with complete bibliographic information, including authors' names, article titles, journal names, year of publication, and DOIs, to ensure transparency and traceability. Furthermore, we applied PRISMA and AMSTAR standards to assess the methodological quality of the included studies, enabling the selection of high-quality research that offers both theoretical insights and methodological guidance for this review.

## 1. SIRT3 and aging

Aging constitutes an evolutionarily conserved biological trajectory associated with progressive attrition of systemic functionality^32^. Cellular senescence, a hallmark of aging, refers to a permanent cessation of cell division induced by various stressors. This state is defined by the senescence-associated secretory phenotype (SASP), accumulation of macromolecular damage, and metabolic dysregulation^33^.

Accumulating evidence indicates that the molecular mechanisms underlying closely align with those governing organismal aging^34^, including chronic inflammation^35^, epigenetic alterations^36^, autophagy malfunction^37^, oxidative stress^38^, metabolic dysfunction^39^, and mitochondrial instability^40^. Molecular investigations have identified SIRT3 as a central coordinator that orchestrates multiple anti-senescence programs across these processes^41^. Through deacetylation of mitochondrial targets, SIRT3 enhances the cellular antioxidant capacity^42^, promotes metabolic flexibility^43^, and reduces ROS accumulation, all of which are critical for minimizing DNA damage and inhibiting the onset of senescence^44^.

Importantly, SIRT3 also regulates canonical markers, such as SA-β-gal and inhibits the p53/p21 axis^45^, thereby delaying the senescence initiation. Moreover, SIRT3 functions as a negative regulator of SASP *via* its control over mitochondrial function^46^. Experimental models have shown that the loss of SIRT3 results in increased expression of SASP components, including TNF-α, IL-1β, and matrix metalloproteinase-9 (MMP9)^47^. Conversely, activation of SIRT3 ameliorates inflammatory tissue damage. Notably, oxidative stress-induced upregulation of microRNA-494-3p (miR-494-3p) leads to decreased SIRT3 levels, establishing a feedback loop that further exacerbates senescence^48^,^49^.

Although cellular senescence and organismal aging are mechanistically intertwined, they present as distinct phenotypic entities^50^. From a translational standpoint, the pleiotropic roles of SIRT3 in fibrosis and apoptosis underscore its potential as a therapeutic target for aging-related pathologies, extending beyond the context of cellular senescence alone. To further explore the molecular crosstalk between SIRT3 and cellular senescence, we focus on the pathways through which SIRT3 exerts its anti-senescence effects. These include the regulation of inflammation, epigenetic modifications, metabolic homeostasis, oxidative stress, autophagy, fibrosis and apoptosis. Together, these interconnected pathways form the molecular foundation for SIRT3's ability to delay aging and alleviate aging-related diseases.

### 1.1 Metabolic regulation

Emerging evidence indicates that SIRT3 acts as a key metabolic regulatory that senses intracellular acetyl-coenzyme A (CoA) and NAD^+^ levels by transducing these cues through the deacetylation of mitochondrial proteins^51^. The downstream targets of SIRT3 span critical mitochondrial pathways, including the tricarboxylic acid (TCA) cycle^52^, amino acid catabolism^53^, lipid β-oxidation^54^, and electron transport chain (ETC)/oxidative phosphorylation (OXPHOS) complexes^55^,^56^.

Mice with SIRT3 knockout exhibited compromised triglyceride catabolism, diminished β-oxidation efficiency, and reduced generation of essential metabolic intermediates such as acetyl-CoA^57^,^58^. Under caloric restriction (CR) or fasting, elevated NAD^+^ levels promote SIRT3 deacetylase activity, optimizing mitochondrial enzymatic efficiency through post-translational modifications^59^. Diminished hepatic SIRT3 expression induces hepatic steatosis by facilitating intracellular triglyceride deposition in response to elevated fatty acid concentrations^60^.

In the context of high-fat diet consumption, preclinical models with pancreatic β-cell-selective SIRT3 knockout exhibited compromised glucose homeostasis, decreased glucose-stimulated insulin secretion, and reduced pancreatic β-cell function^61^. Transcriptomic profiling further revealed that β-cell-selective SIRT3 knockout mediates insulin secretion and liver lipid homeostasis through a serotonin-dependent mechanism^62^.

**Figure 1:**
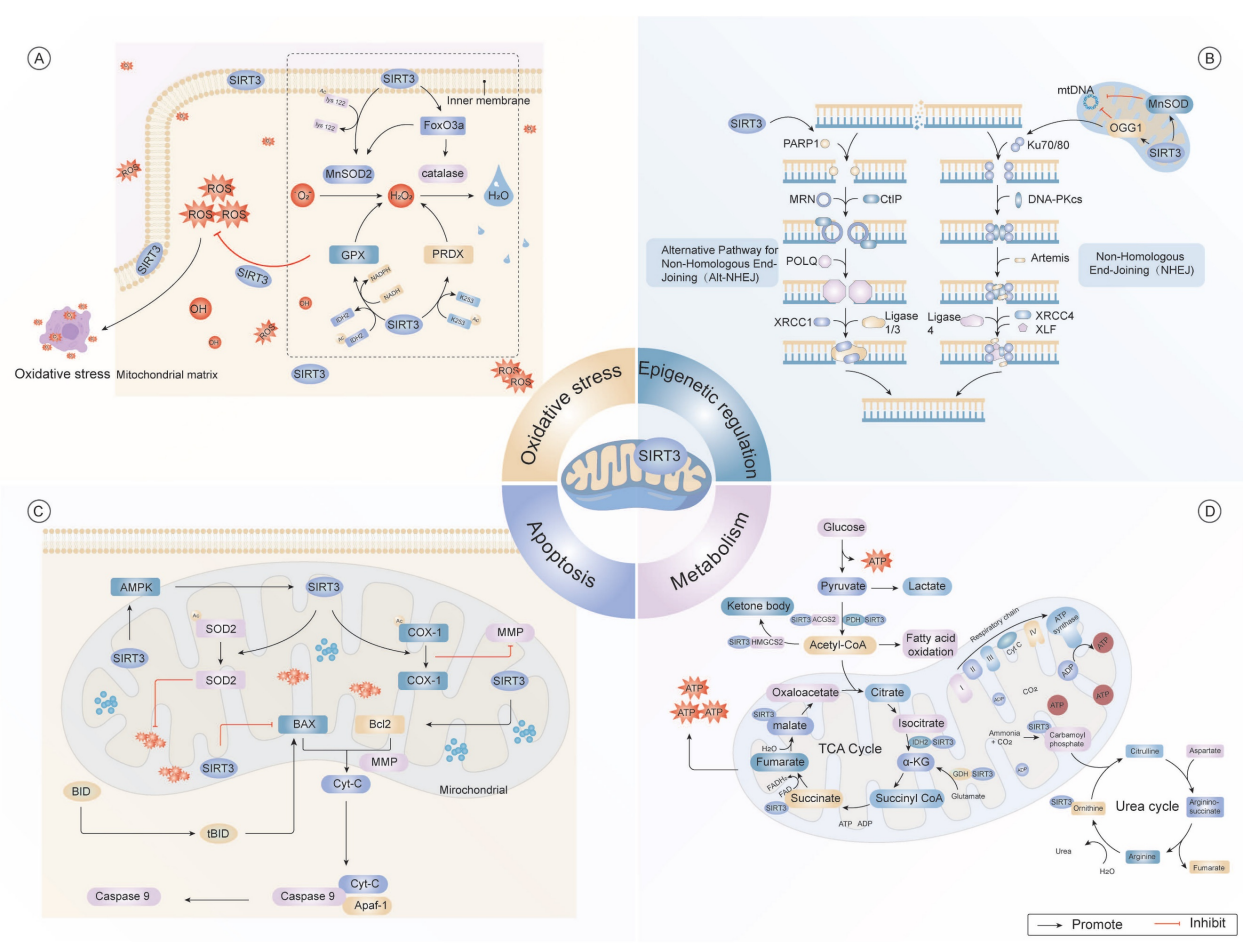
** Multidimensional regulatory network of SIRT3 in cellular homeostasis. (A)** As a central redox regulator, SIRT3 orchestrates mitochondrial antioxidant defense through dual mechanisms. It regulates key antioxidant enzymes, including MnSOD2 and CAT, to counteract oxidative damage induced by ROS. Furthermore, SIRT3 modulates the activities of GPX and PRDX, thereby maintaining cellular redox homeostasis. **(B)** SIRT3 contributes to the regulation of epigenetic processes, particularly in DNA repair mechanisms *via* the NHEJ pathway. It interacts with critical DNA repair proteins, such as PARP1, Ku70/80, XRCC1, and DNA ligase 4, and facilitates the repair of mtDNA damage. **(C)** SIRT3 modulates apoptotic commitment through dynamic acetylation networks. SIRT3 regulates mitochondrial dynamics by deacetylating Bcl-2, thus preventing mitochondrial outer membrane permeabilization. Additionally, SIRT3 delays Cyt-C release kinetics the subsequent activation of caspases, thereby modulating apoptotic signaling pathways. **(D)** SIRT3 integrates metabolic flux through substrate channeling, exerting significant effects on the TCA cycle and promoting fatty acid oxidation. It regulates critical enzymes, such as AMPK, SOD2, and acetyl-CoA, and also modulates glucose metabolism by controlling the conversion of pyruvate to lactate. Moreover, SIRT3 supports the production of ketone bodies, which are essential for maintaining energy balance under conditions of metabolic stress.

SIRT3 also regulates succinate dehydrogenase (SDH/complex II), a critical acetylation node within the mitochondrial matrix, which mediates crosstalk between metabolic flux control and mitochondrial energy transduction systems^63^. Through the modulation of SDH enzymatic activity, SIRT3 fine-tunes substrate utilization efficiency while maintaining electron transfer chain synchronization, thus preserving metabolic homeostasis^64^. Furthermore, experimental data have demonstrated that shRNA-induced SIRT3 knockdown significantly attenuates the cytoprotective effects of honokiol (HKL) on mitochondrial biogenesis, OXPHOS capacity, and bioenergetic output, underscoring its pivotal role in maintaining mitochondrial biogenesis and bioenergetics^65^ (Fig. 1).

These findings established SIRT3 as a crucial modulator of metabolic homeostasis through its multifaceted catalytic functions. Future research should focus on delineating the disease-specific mechanisms of SIRT3 and evaluating its therapeutic utility in metabolic and age-related diseases.

### 1.2 Oxidative stress

Oxidative stress represents a pathological condition defined by a systemic imbalance in which overproduction of reactive oxygen species (ROS) that overwhelms the detoxification potential of endogenous cytoprotective machinery^66^. Such perturbations initiate structural alterations in biomacromolecules, thereby disrupting cellular function and promoting aging-related pathologies^67^.

Previous studies have established that SIRT3 exerts potent deacetylase activity on ETC subunits and enzymes involved in oxidative stress response activity, serving as a critical factor in various underlying regulatory mechanisms^68^. Furthermore, SIRT3 augments ROS scavenging while maintaining organelle homeostasis, conferring cytoprotective effects against oxidative stress^69^. Experimental evidence has demonstrated that mitochondrial antioxidant enzymes display altered lysine acetylation modification in SIRT3-deficient conditions, exhibiting pronounced acetylation leading to enzymatic dysfunction, which exacerbates ROS generation and aggravates oxidative stress-induced damage^70^.

Emerging research has underscored the vital role of SIRT3 in mitigating oxidative stress through precise regulation of core antioxidant enzymes, including manganese superoxide dismutase 2 (MnSOD2), glutathione peroxidase, and isocitrate dehydrogenase 2 (IDH2)^71^. Under caloric restriction, SIRT3-mediated deacetylation dynamically regulates mitochondrial IDH2 activation, culminating in elevated NADPH biosynthesis^72^, prevents ETC overload and subsequent excessive ROS generation and potentiates mitochondrial antioxidant capacity, ultimately attenuating oxidative stress^73^,^74^. Notably, SIRT3 optimizes mitochondrial ROS clearance by deacetylating SOD^75^,^76^. Furthermore, SIRT3 enhances MnSOD activity through deacetylation, amplifying endogenous antioxidant responses^77^. Additionally, the SIRT3/forkhead box O3a (FOXO3a) signaling axis triggers mitochondrial DNA (mtDNA) expression, thereby alleviating oxidative damage^78^ (Fig. 1).

Hence, SIRT3 maintains mitochondrial redox homeostasis and attenuates oxidative stress-associated cellular damage through epigenetic regulation of antioxidant defense systems. The therapeutic potential of targeting SIRT3 in aging-related metabolic diseases and neurodegenerative disorders presents a promising avenue for future research.

### 1.3 Epigenetic regulation

Epigenetic regulation refers to a set of dynamic and reversible modifications spanning genomic DNA and nucleoprotein assemblies, orchestrated through intricate interactions between sequence-specific transcription factors and various epigenetic modifier complexes^79^. Accumulating evidence positions SIRT3 as a novel chromatin-modifying enzyme, exerting tripartite regulatory control over DNA methylation patterns, covalent histone modifications, and nucleosome remodeling^80^.

Mechanistically, SIRT3 modulates the enzymatic catalytic function of 8-oxo guanine DNA glycosylase-1 (OGG1) through lysine deacetylation, potentiating mitochondrial DNA damage remediation^81^. It also promotes the transcriptional activity of FOXO3a through deacetylation, thereby increasing its DNA-binding affinity and enhancing the expression of genes involved in cellular stress-responsive^82^. Notably, SIRT3 plays a critical geroprotectvive factor that maintains heterochromatin integrity in hMSCs, thereby postponing replicative senescence progression^83^. Furthermore, SIRT3 forms macromolecular assemblies with nuclear lamina constituents and heterochromatin-associated proteins, supporting chromatin compaction and genomic stability maintenance^84^.

The absence of SIRT3 triggers structural abnormalities in the nuclear envelope, disarray of heterochromatin organization, and reactivation of previously silenced repetitive sequences, collectively accelerating premature cellular aging^85^,^86^. Restoration of SIRT3 expression in deficient cells has been shown to re-establish chromatin organization and mitigate senescence-associated phenotypes^87^.

Advances in high-resolution chromatin profiling have illuminated previously uncharacterized mechanisms by which SIRT3 maintains genomic integrity^88^. Beyond its canonical deacetylase activity, SIRT3 possesses substantial lysine decrotonylase capacity^89^, selectively removing crotonyl groups from specific histone substrates^90^. This dynamic regulation of histone crotonylation adds a new dimension to SIRT3's epigenetic repertoire, particularly in modulating gene expression plasticity under both homeostatic and stress conditions^91^.

Moreover, SIRT3 undergoes stress-responsive chromatin redistribution^92^, influencing higher-order chromatin topology and initiating protective transcriptional programs in response to genotoxic stimuli^93^. Disruption of SIRT3 impairs the chromatin-silencing machinery, leading to altered transcriptional landscapes and compromised chromatin homeostasis^94^. Importantly, SIRT3 also facilitates DNA double-strand break repair by enhancing the recruitment of 53BP1, thus promoting the efficiency of the non-homologous end joining (NHEJ)^95^.

In the context of viral infection, SIRT3 dynamically modulates the transcriptional output and replicative capacity of viral genomes by guiding histone methyltransferase complexes to viral episomes^96^. Exogenous SIRT3 overexpression significantly attenuates virus gene expression, whereas deletion potentiates viral replication efficiency^97^. Additionally, SIRT3 decreases H3K27 crotonylation at metastasis-associated loci, such as ETS1, effectively constraining neoplastic dissemination^98^,^99^ (Fig. 1).

Taken together, these findings portray SIRT3 as a critical integrator of chromatin signaling pathways, positioned at the interface of epigenetic regulation and genome surveillance. Future investigations should aim to delineate the disease-specific chromatin landscapes modulated by SIRT3, with a particular focus on its roles in tumor biology, neurodegenerative disorders, and viral pathogenesis.

### 1.4 Apoptosis

Apoptosis exhibits paradoxical roles in maintaining organismal homeostasis^100^. As a bifunctional regulator of cellular dynamics, apoptosis maintains tissue homeostasis through precisely orchestrated mitochondrial^101^ and endoplasmic reticulum stress responses pathways^102^.

These processes yield divergent outcomes depending on whether the cellular environment is physiological or pathophysiological^103^.

In the context of senescent cells, sustained low-grade stress induces resistance to apoptosis signals, fostering a pro-inflammatory and senescence-associated phenotype, which accelerates systemic aging^104^,^105^.

Emerging evidence highlights the context-dependent manner of SIRT3 in modulating apoptotic responses^106^. Under stress conditions, SIRT3 predominantly exerts anti-apoptotic effects by orchestrating apoptotic regulatory hubs, such as the GSK-3β/Bax, Bax/Bcl-2, and caspase-9 pathways^107^,^108^. Through these interactions, SIRT3 safeguards mitochondrial integrity and promoting cell viability^109^. Notably, the AMPK/SENP1/SIRT3 axis governs mitochondrial apoptosis *via* SOD2 deacetylation, thereby mitigating oxidative stress-induced apoptosis^110^.

Conversely, under certain pathological conditions such as malignancy, SIRT3 exerts pro-apoptotic effects by facilitating mitochondrial apoptotic signaling, thereby impeding tumor proliferation and viability^111^. In sepsis models, genetic ablation of SIRT3 has been associated with intensified apoptotic responses, as evidenced by increased Bax and caspase-3 levels alongside reduced Bcl-2 expression^112^,^113^. This pro-apoptotic function is further potentiated by FOXO1 deacetylation, which upregulates transcription of apoptosis-promoting genes^114^,^115^. Moreover, non-coding RNAs, such as miR-297^116^ and miR-421^117^, have emerged as upstream modulators of SIRT3, modulating its expression and consequently influencing apoptosis-related pathways (Fig. 1).

Collectively, these insights underscore the dualistic and context-dependent roles of SIRT3 in apoptosis regulation. Serving as a pivotal node in apoptotic signaling networks, SIRT3 orchestrates cellular fate decisions with implications across aging and disease states. Future investigations should aim to dissect the tissue-specific functions of SIRT3, explore its molecular crosstalk with other apoptotic regulators, and evaluate its potential as a therapeutic target in aging-related disorders such as cancer.

### 1.5 Fibrosis

Fibrosis represents a hallmark of aging that arises from a progressive decline of tissue restorative and regenerative competence^118^,^119^. In senescent organs, injury-induced signals increasingly favor pro-fibrotic cascades rather than activating regenerative programs, leading to excessive extracellular matrix (ECM) deposition and increased tissue rigidity^120^,^121^. This maladaptive remodeling disturbs normal tissue architecture and function, ultimately driving multi-organ dysfunction in older individuals^122^.

Recent investigations have identified SIRT3 as a critical suppressor of fibrogenesis, operating at the interface between mitochondrial homeostasis and nuclear transcriptional regulation^123^. Across a range of experimental fibrosis models, including peritoneal, cardiac, pulmonary, and hepatic fibrosis, SIRT3 consistently act as a central suppressor of fibrotic progression^124^. Mechanistically, SIRT3 mediates its anti-fibrotic effect through the targeted deacetylation of glycogen synthase kinase 3β (GSK-3β) at Lys15, effectively preventing Smad3 transcriptional complex formation and subsequent fibrogenic programming^125^. In cardiomyocytes, SIRT3 also attenuates fibrosis by inhibiting the FOS/AP-1^126^ and transcription 3 (STAT3)/NFATc2 signaling pathways^125^. Conversely, genetic deletion of SIRT3 exacerbates susceptibility to fibrosis^127^, particularly in the myocardium, where its absence leads to increased GSK-3β acetylation^128^ and hyperactivation of the TGF-β signaling axis^129^. These alterations culminate in enhanced transcription of ECM-related genes.

Notably, the pro-fibrotic consequences of SIRT3 deficiency are not confined to cardiac tissue. Similar signaling disruptions have observed in renal and pulmonary fibrosis models, where SIRT3 restoration effectively reverses fibrotic pathology by rebalancing mitochondrial redox states and repressing Smad3-mediated transcription activity^130^.

**Figure 2:**
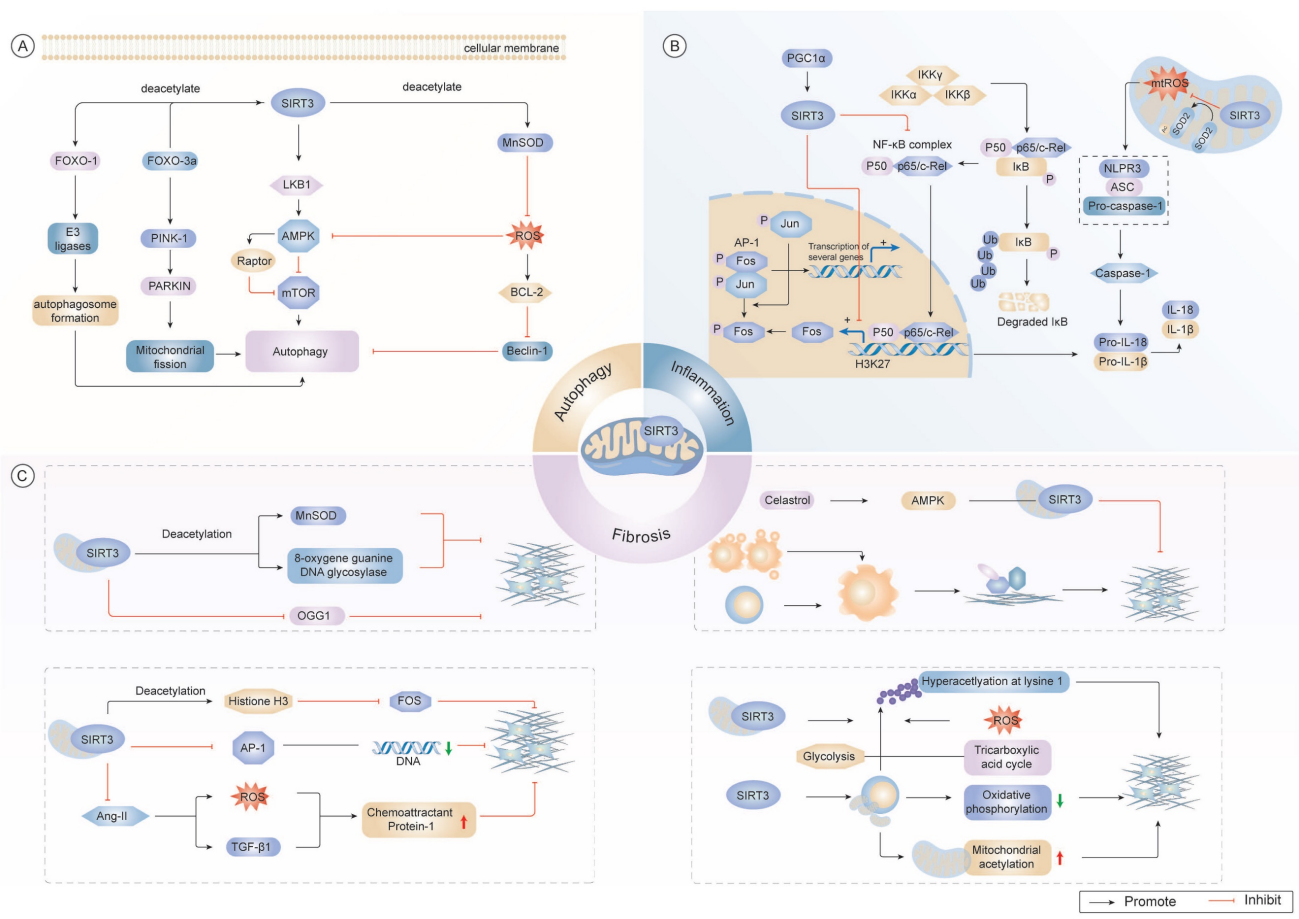
** Multifaceted regulatory mechanisms of SIRT3 in cellular homeostasis. (A)** SIRT3 regulates autophagy through the deacetylation of FOXO1 and FOXO3a, which interact with E3 ligase and PARKIN to promote mitochondrial fission. Additionally, SIRT3 activates the LKB1/AMPK axis, thereby modulating autophagy *via* mTOR inhibition and Raptor activation, maintaining mitochondrial homeostasis and cellular integrity. Furthermore, SIRT3 influences mitochondrial dynamics by promoting the expression of MnSOD, thus alleviating oxidative damage caused by ROS. **(B)** SIRT3 suppresses pro-inflammatory signaling by deacetylating transcription factors NF-κB and AP-1, thereby attenuating NLRP3 inflammasome assembly and subsequent interleukin release. SIRT3 modulates the chemotactic proteins levels such as TGF-β1 in response to stress signals such as Ang-II. **(C)** In fibrosis, SIRT3 regulates the expression of fibrotic markers by deacetylating histone H3, thereby influencing gene transcription. It affects pathways related to extracellular matrix remodeling, including directly modulating TGF-β1 expression, a key driver of fibrosis. Metabolically, SIRT3 coordinates glycolysis, TCA cycle flux, and oxidative phosphorylation. by deacetylating key enzymes such as MnSOD, ensuring efficient energy production. By dynamically regulating mitochondrial protein acetylation, SIRT3 enables adaptive metabolic reprogramming during cellular stress.

In pulmonary tissue, SIRT3 overexpression has been shown to mitigate bleomycin-induced fibrosis, primarily by preserving mtDNA integrity and suppressing TGF-β1-dependent signaling pathways^131^-^133^. In hepatic fibrosis models, SIRT3 has been implicated in mediating the anti-fibrotic effects of withaferin A^134^, an effect that is abolished in SIRT3-knockout mice. This finding reinforces the indispensable role of SIRT3 as a molecular effector of pharmacological anti-fibrotic agents^126^ (Fig. 2).

Collectively, these findings establish SIRT3 as a master regulatory nexus in the pathogenesis of fibrosis across multiple organ systems. By integrating mitochondrial integrity, redox homeostasis, and nuclear transcriptional control, SIRT3 orchestrates protective responses against fibrotic remodeling. Moving forward, research should focus on elucidating the organ-specific and context-dependent mechanisms by which SIRT3 modulates fibrosis. Particular attention should be directed toward the interplay between SIRT3's mitochondrial and nuclear activities during the transition from tissue injury to fibrotic remodeling, as well as its potential crosstalk with ECM-sensing mechanisms. These insights may pave the way for developing targeted therapeutic interventions aimed at modulating SIRT3 activity in fibrotic disease.

### 1.6 Inflammation

Low-grade inflammatory states have emerged as pathognomonic features of organismal senescence and primary drivers in geriatric pathobiology^135^. SIRT3 serves as a critical molecular determinant by modulating aging-related inflammation through its regulation of mitochondrial homeostasis and inflammatory signaling pathways^136^.

Studies revealed greater ROS accumulation, amplified NLRP3 inflammasome oligomerization, and exacerbated mitochondrial structural abnormalities in SIRT-knockout murine models compared with the findings in their wild-type counterparts^124^,^137^. Conversely, SIRT3 overexpression attenuates inflammation by inhibiting IκBα phosphorylation^138^ and modulating the nuclear factor kappa-light-chain-enhancer of activated B cells (NF-κB)/TGF-β1/Smad axis^139^, thereby suppressing NLRP3 inflammasome formation and subsequent inflammatory responses^116^,^140^.

Notably, SIRT3 activation consistently correlates with reduced expression of pro-inflammatory cytokines, such as TNF-α, IL-6, and MIP-2, as well as diminished polymorphonuclear leukocyte infiltration in various preclinical models^141^,^142^. In murine models with chronic sodium overload, SIRT3 overexpression induced persistent disruption of chronic sodium overload, SIRT3 overexpression disrupted immune cell migration by modulating key metabolic and immune signaling pathways^143^. These anti-inflammatory effects were sustained even in the absence of continued dietary interventions, indicating that SIRT3 mediates durable transcriptional reprogramming *via* NF-κB and STAT3 suppression^144^,^145^ (Fig. 2).

Taken together, these findings position SIRT3 as a mitochondrial checkpoint in the regulation of inflammation during aging. Future investigations should aim to elucidate the mechanisms by which SIRT3 interfaces with tissue-specific immune circuits and determine whether pharmacologic activation of SIRT3 can confer resilience against aging-related inflammatory disorders such as atherosclerosis, neuroinflammation, and sarcopenia. Additionally, further research is warranted to uncover the upstream regulatory networks that govern SIRT3 activity under conditions of inflammatory stress.

### 1.7 Autophagy

Autophagy, a phylogenetically conserved lysosomal clearance mechanism, sustains proteostasis *via* the selective elimination of dysfunctional organelles and misfolded protein aggregates^146^. SIRT3 has emerged as a pivotal regulator of autophagic flux dynamics, modulating evolutionarily conserved signaling pathways, including the AMPK/mTOR and FOXO3a pathways^147^. Genetic ablation of SIRT3 impairs autophagic signaling, notably disrupting AMPK/mTOR-mediated transduction concomitant with the paradoxical activation of glutathione peroxidase 4 activity, which suppresses autophagy initiation^148^.

Mechanistically, SIRT3 facilitates mitochondrial quality control *via* AMPK/ULK1 phosphorylation while antagonizing mTOR-mediated autophagic repression, thereby establishing dual checkpoints for organelle surveillance^149^. Moreover, a positive feedback loop exists in which autophagy activation induces SIRT3 expression *via* FOXO3a-mediated transcriptional programming, thereby promoting PTEN-induced kinase (PINK1)/Parkin-mediated mitochondrial quality control to reduce mtROS levels and restore hematopoietic stem cell repopulation efficiency through the enhanced lysosomal clearance of damaged mitochondria^150^-^152^(Fig. 2).

Taken together, these findings underscore SIRT3 as a central integrator of the autophagy-lysosome network, orchestrating mitochondrial fidelity, proteostasis, and stem cell maintenance during aging. Future investigations should focus on elucidating the tissue-specific roles of SIRT3 in autophagy regulation and exploring whether pharmacological modulation of SIRT3 can be leveraged to enhance autophagic function in aging-related degenerative conditions.

## 2. The role of SIRT3 in aging-related diseases

Aging-related disorders arise within tissue microenvironments due to chronic inflammation, stem cell exhaustion, and structural remodeling associated with aging^153^. As an integrative coordinator of senescence mechanisms, SIRT3 has emerged as a pleiotropic safeguard against aging-related pathological progression through multimodal cytoprotective mechanisms^55^.

### 2.1 SIRT3 and neurodegenerative diseases

**Figure 3:**
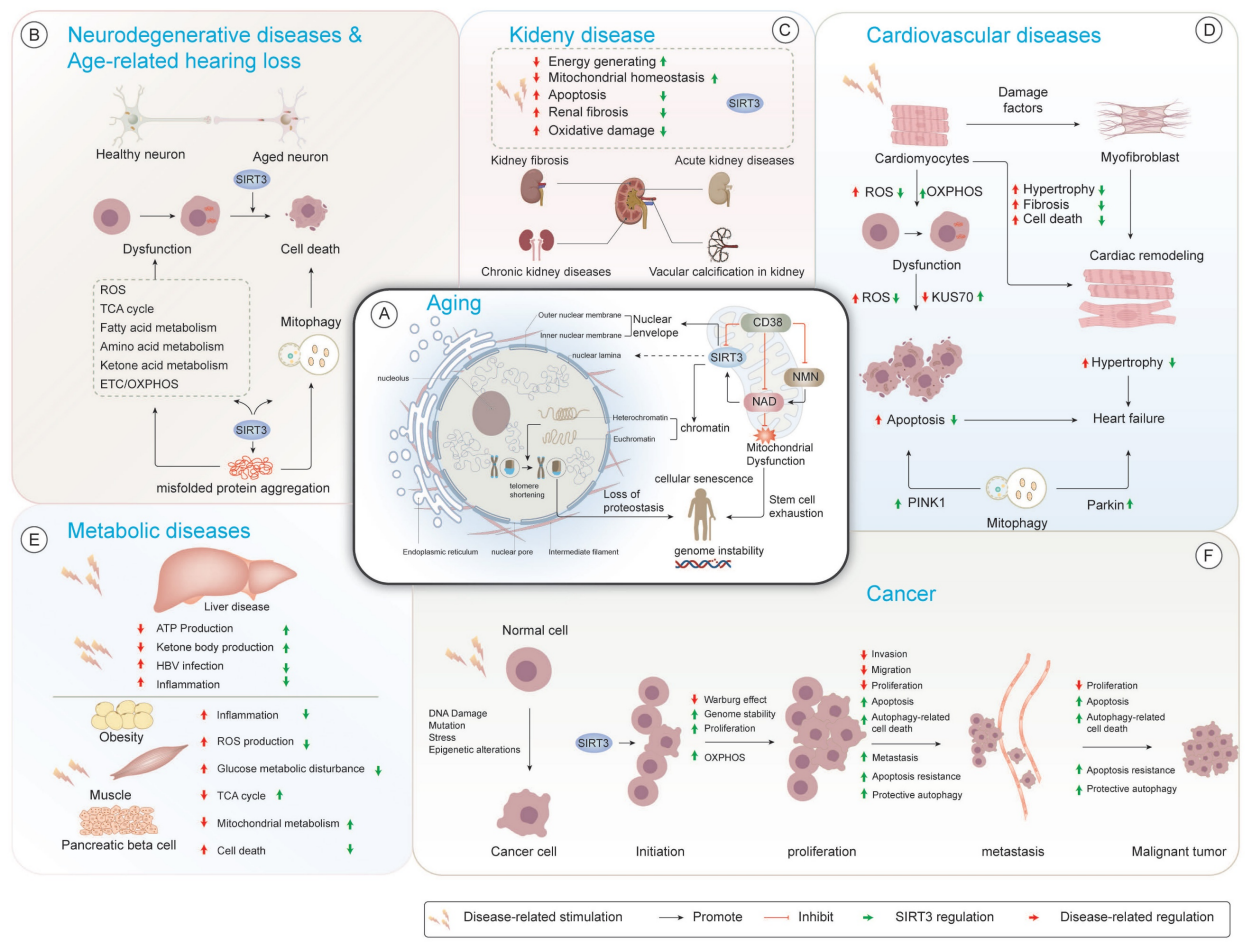
** Regulatory mechanisms of SIRT3 in aging-related pathologies. (A) Cellular senescence mechanisms.** Aging is associated with mitochondrial dysfunction and nuclear instability. SIRT3 regulates essential cellular processes involved in the maintenance of nuclear architecture, chromatin remodeling, and proteostasis, thereby influencing cellular senescence, stem cell exhaustion, and genome instability. **(B) Neurodegenerative diseases and age-related hearing loss.** SIRT3 exerts regulatory control over mitochondrial dynamics through the precise modulation of TCA cycle intermediates, lipid catabolism, and amino acid metabolic pathways, effectively counteracting ROS-induced oxidative stress. Additionally, SIRT3 mitigates the aggregation of misfolded proteins, which is essential for preserving neuronal function during aging. **(C) Renal pathophysiology.** Under chronic renal pathological conditions, SIRT3 demonstrates nephroprotective effects by enhancing mitochondrial bioenergetics, attenuating oxidative damage, and improving cellular energy transduction mechanisms. Its role in controlling renal fibrosis and apoptosis is crucial for preventing acute kidney injury and mitigating kidney dysfunction. **(D) Cardiovascular pathology.** SIRT3 protects cardiomyocytes by modulating ROS levels, oxidative phosphorylation, and KUS70 expression. SIRT3's cardioprotective actions encompass suppression of pathological hypertrophy and apoptotic signaling *via* selective mitochondrial quality control mechanisms, particularly through PINK1-mediated autophagic pathways. **(E) Metabolic dysregulation.** SIRT3 serves as a master regulator of bioenergetic processes, including ATP synthesis, gluconeogenic pathways, and ketone body production, particularly in metabolic disorders including obesity and non-alcoholic fatty liver disease. Its ability to mitigate ROS production and restore mitochondrial function in muscle and pancreatic β-cells underscores its importance in metabolic homeostasis. **(F) Oncogenic processes.** SIRT3 is integral to cancer progression, as it modulates cancer-specific metabolic adaptations including aerobic glycolysis (Warburg effect) and glutaminolysis and increases oxidative phosphorylation efficiency through complex I/III activity modulation. SIRT3 orchestrates key cell fate decisions *via* cell proliferation, apoptosis, and autophagy, thereby modulating cancer cell survival and metastasis.

Neurodegenerative disorders represent a diverse array of pathophysiological entities defined by the time-dependent degeneration of specific neuronal populations within functionally interconnected neural networks^154^. This disease category is clinically exemplified by four principal clinical-pathological entities: Alzheimer's disease (AD), Parkinson's disease (PD), amyotrophic lateral sclerosis (ALS), and Huntington's disease (HD)^155^. Quantitative proteomic analyses demonstrated evolutionarily conserved maintenance of cerebral SIRT3 expression trajectories throughout ontogenic development and senescence^43^,^156^, thereby underscoring its regulatory nexus within neurodegenerative pathophysiology^157^ (Fig.3).

#### 2.1.1 AD

AD, the predominant aging-related neurodegenerative disorder, is pathologically marked by the extracellular deposition of amyloid-beta (Aβ) plaques and intraneuronal neurofibrillary tangles composed of hyperphosphorylated tau proteins^158^,^159^, which synergistically drive irreversible mnemonic deterioration and cognitive decline^160^. Among the sirtuin family of proteins, SIRT3 is the most abundantly expressed in the central nervous system^161^, but its mRNA expression exhibits a progressive decline spanning from clinical specimens to experimental rodents^162^-^164^. Recent findings revealed that SIRT3 plays a multifaceted and context-dependent neuroprotective role in the context of AD. SIRT3 regulates critical processes such as neurogenesis, neuroinflammation, and mitochondrial homeostasis^165^-^167^, thus contributing to its neuroprotective effects.

Notably, translational research has demonstrated an inverse association between SIRT3 levels and tau protein deposition^168^, indicating a potential modulatory role of SIRT3 in tauopathies. Mechanistically, SIRT3 has been shown to enhance mitochondrial bioenergetics, which are disrupted by Aβ toxicity^169^, partially through the deacetylation of mitochondrial p53 at Lys320^170^. This modification is crucial for maintaining energy homeostasis and neuronal survival. Meanwhile, SIRT3 knockout exacerbates synaptic degeneration by disrupting mitochondrial energy regulation^171^.

In addition to its role in metabolic processes, SIRT3 also plays a critical role in regulating neuronal oxidative stress by activating MnSOD and stabilizing mitochondrial dynamics through key post-translational modifications^163^. Furthermore, SIRT3 contribute to mitochondrial quality control through the PINK1/Parkin pathway, facilitating the mitophagic clearance of damaged mitochondria and mitigating both amyloidogenesis and tau pathology^172^. Interestingly, SIRT3 also modulates neuropeptide signaling pathways. Its interaction with pituitary adenylate cyclase-activating polypeptide enhances neuroprotective effects by alleviating Aβ toxicity^173^, indicating that SIRT3 can bridge neurotrophic and metabolic signaling in the aging brain.

Although the involvement of SIRT3 in the regulation of AD pathology regulation is becoming increasingly evident, the precise upstream cues that drive its downregulation in AD remain poorly understood. Future studies should investigate whether early exposure to Aβ directly impairs SIRT3 transcription or post-translational stability and whether glial SIRT3 is involved in modulating microglial or astrocytic responses during neuroinflammation. Additionally, the therapeutic potential of SIRT3 activation warrants further investigation. Lastly, the possibility of SIRT3 serving as a biomarker for early mitochondrial dysfunction in the progression of AD is an exciting avenue for future research.

#### 2.1.2 PD

PD represents a multifactorial neurodegenerative condition with multifactorial etiology, pathologically defined by selective degeneration of dopaminergic neurons in the nigrostriatal pathway^174^, leading to the cardinal motor symptom triad of bradykinesia, resting tremor, and rigidity^175^. Accumulating evidence indicates that mitochondrial dysfunction, oxidative stress, and chronic neuroinflammation are central contributors to PD pathogenesis. Among the key regulators of these processes, SIRT3 has emerged as a critical upstream regulator^25^,^177^.

Preclinical investigations have shown that elevated SIRT3 expression confers substantial neuroprotection to dopaminergic (DA) neurons through multiple converging mechanisms^178^. One critical pathway involves the deacetylation of dynamin-related protein 1 (DRP1), a mitochondrial fission protein implicated in the pathological fragmentation of mitochondria in PD^176^. SIRT3-mediated modulation of DRP1 activity restores mitochondrial dynamics, protects against DA neuronal loss, and ameliorates motor deficits in murine models^179^. Concurrently, SIRT3 enhances mitochondrial resilience by scavenging ROS, preserving ETC integrity, and promoting mitochondrial autophagy, thereby attenuating neurodegeneration in midbrain dopaminergic populations^180^. Mechanistic investigations further elucidated that SIRT3 governs mitochondrial biogenesis by activating the peroxisome proliferator-activated receptor gamma coactivator 1-alpha (PGC-1α) transcriptional activation, which in turn maintains mitochondrial DNA integrity in murine models^181^.

Beyond its roles in mitochondrial homeostasis, SIRT3 also contributes to the clearance of misfolded α-synuclein aggregates, a pathological hallmark of PD^182^. Furthermore, SIRT3 mitigates neuroinflammation by suppressing NLRP3 inflammasome activation^183^. Together, these diverse actions highlight the translational potential of targeting SIRT3 in PD therapy.

Despite compelling evidence from preclinical models supporting SIRT3's neuroprotective effects, its precise role in the initiation and early progression of PD remains unclear. Future research is required to determine whether alterations in SIRT3 expression precede dopaminergic neurodegeneration and to delineate its specific functions across neuronal and glial cell populations. Moreover, the development of selective, brain-permeable SIRT3 activators could open promising avenues for translational intervention in PD.

#### 2.1.3 ALS

ALS represents a fatal neurological deterioration featuring the concurrent involvement of both upper motor neurons and lower motor neurons, progressing to neuromuscular junction disintegration, progressive myofiber degeneration, and premature fatality^184^.

Mechanistic studies revealed that dysregulation of SIRT3 plays a significant role in ALS pathogenesis. Specifically, loss of SIRT3 activity promotes aberrant mitochondrial protein hyperacetylation, thereby compromising OXPHOS complex stoichiometry and ETC capacity^185^. Pharmacological potentiation of SIRT3 by nicotinamide (NAM)^186^ has been shown to preserve neuronal ultrastructural integrity and viability in ALS models^187^. Notably, overexpression of SIRT3 in mutant SOD1 (G93A)-induced ALS models, ameliorates disease phenotypes^188^, suggesting a potential causal relationship between SIRT3 function and ALS progression.

Beyond its neuronal effects, SIRT3 dysregulation disrupts the fidelity of NAD^+^ salvage pathways within striated muscle microenvironments, perpetuating metabolic disturbances that exacerbate neuromuscular degeneration in ALS progression^25^. Collectively, these findings underscore the pivotal role of SIRT3 in modulating mitochondrial metabolism and preserving neuromuscular function in the context of ALS.

Despite robust evidence from preclinical models supporting the neuroprotective role of SIRT3, its temporal dynamics and cell-type specific actions in ALS remain insufficiently defined. Future research should clarify whether SIRT3 exerts distinct effects in motor neurons versus skeletal muscle, and determine whether therapeutic activation of SIRT3 yields greater benefit in early versus late disease stages of the disease. Furthermore, validation in patient-derived tissues will be critical to assess the clinical relevance of SIRT3 as a potential biomarker or therapeutic target in ALS.

#### 2.1.4 HD

HD is an autosomal dominant trinucleotide repeat disorder with the neuropathological hallmark of a distinct symptom triad featuring hyperkinetic movement disorders including choreodystonic movements and gait ataxia, progressive cognitive impairment, and neuropsychiatric manifestations^189^,^190^. Post-mortem analyses of human brain tissue and investigations using transgenic animal models have identified aberrant SIRT3 expression as a consistent molecular signature associated with HD pathology^191^.

Emerging evidence suggests that SIRT3 plays a protective role in mitigating the pathogenic effects of mutant huntingtin protein. Overexpression of SIRT3 has been shown to ameliorate clinical symptoms and support the survival of striatal neurons^192^, whereas SIRT3 deficiency aggravates mitochondrial impairment and heightens neuronal susceptibility to excitotoxic damage^193^. Mechanistically, SIRT3 enhances mitochondrial bioenergetics, reduces oxidative stress, and prevents glutamate-induced apoptosis. These protective effects are mediated, at least in part, by regulation of NAD⁺ metabolism^125^,^194^ and activation of AMPK phosphorylation, which promotes PGC-1α-dependent transcriptional programs to sustain redox homeostasis and neuronal energy metabolism^195^. Furthermore, genetic ablation of SIRT3 exacerbates oxidative damage markers, whereas pharmacological redox modulators restore physiological SIRT3 levels *via* nuclear factor erythroid 2-related factor 2 (Nrf2)-mediated transcriptional feedback loops^68^.

In addition to metabolic regulation, SIRT3 also maintains mitochondrial dynamics by suppressing fission regulators, such as Fis1 and Drp1, thereby preserving mitochondrial morphology and promoting efficient axonal transport^196^,^197^. In *Drosophila melanogaster* HD models, transgenic expression of the SIRT3 ortholog dSirt2 significantly attenuated neuropil degeneration and prolonged organismal longevity^193^, reinforcing its conserved neuroprotective functions.

Despite compelling preclinical findings, the spatiotemporal dynamics and cell-type specificity of SIRT3 activity in HD remain poorly understood. Future research should investigate whether modulation of SIRT3 can delay disease onset or influence progression *in vivo*, and evaluate its potential synergistic effects with existing mutant huntingtin-lowering therapeutic strategies. The development of selective SIRT3 agonists with optimized blood-brain barrier permeability holds promise as a novel approach for HD management.

### 2.2 SIRT3 and cardiovascular disease (CVD)

CVD is the principal contributor to global mortality, imposing a substantial socioeconomic burden with elevated healthcare expenditures and workforce attrition in aging demographics^198^. Nevertheless, the geriatric-specific pathophysiology mechanisms underlying CVD progression persists as a poorly characterized domain.

Growing evidence implicates that SIRT3 as a key contributor to aging-related CVD, whereas SIRT3 upregulation by exogenous factors ameliorates disease progression in preclinical models^199^. Mechanistically, SIRT3 exerts pleiotropic cardioprotective effects, including the regulation of mitochondrial protein deacetylation, attenuation of oxidative stress, and modulation of ECM remodeling^200^. These functions are essential for preserving cardiovascular homeostasis and tissue integrity during aging. Taken together, these findings establish the therapeutic promise of SIRT3 as a potential molecular target for aging-related CVD pathologies (Fig.3).

#### 2.2.1 SIRT3 and atherosclerosis

Atherosclerosis is a multifactorial vascular disease arises from dynamic interactions among chronic vascular inflammation, endothelial cell (EC) dysfunction, and dysregulated lipid homeostasis^201^. As a crucial mitochondrial deacetylase, SIRT3 deficiency increases oxidative stress and promotes plaque formation, thereby accelerating atherosclerosis pathological process^202^.

Mechanistically, SIRT3 regulates lipid metabolism through multiple interrelated signaling pathways^203^. Specifically, SIRT3 promotes AMPK activation and modulates the activity of uncoupling protein-1 (UCP1), thereby suppressing ox-LDL-induced foam cell formation^204^-^207^. Moreover, gut microbiota-derived metabolites can influence the SIRT3/SOD2/FOXO3A axis, which antagonizes lipogenic programming governed by the SREBP1c/FAS/DGAT2 cascade^208^. This indicates a functional link between SIRT3 activity and host-microbiome interactions in atherosclerosis. However, paradoxical findings from LDL receptor-knockout murine models featured no significant alteration in atherosclerotic lesion development upon SIRT3 deletion^209^, suggesting that the role of SIRT3 in atherosclerosis may be a context-dependent manner. Beyond lipid regulation, SIRT3 plays a pivotal role in maintaining EC function by preserving mitochondrial integrity *via* the SIRT3/ATG5 axis, thereby reducing ROS levels and sustaining nitric oxide bioavailability^210^. The absence of SIRT3 in EC-specific knockout models leads to exaggerated NLRP3 inflammasome activation and endothelial dysfunction^211^, thereby fostering a pro-inflammatory and pro-atherogenic phenotype.

Dietary intervention studies employing hyperlipidemic regimens in murine models demonstrated that SIRT3 deficiency further amplifies monocyte infiltration and cytokine production, primarily *via* dysregulation of NF-κB signaling^208^,^212^. At the molecular level, SIRT3-induced FOXO3a deacetylation and subsequent target catalase (CAT) activation help counteract oxidative stress^213^, reinforcing the anti-inflammatory function of SIRT3. Concurrently, the regulatory effects of SIRT3 on autophagic progress influence both foam cell formation and inflammation responses, key features of atherosclerotic disease progression^214^.

Collectively, these findings validate SIRT3 as a therapeutic target for vascular remodeling, thereby positioning pharmacological modulation of this deacetylase as a promising therapeutic strategy targeting vascular pathologies.

#### 2.2.2 SIRT3 and heart failure (HF)

Chronic cardiac ischemia induced by diverse pathological conditions, such as hypertrophic cardiomyopathy, ischemic myocardial injury, and atherosclerotic coronary obstruction, progressively evolves into HF, a terminal phase of cardiovascular disorders responsible for substantial global morbidity and mortality^215^,^216^. Accumulating evidence indicates that cardiac SIRT3 expression is consistently decreased during HF pathogenesis, paralleling hyperacetylation-induced mitochondrial proteome dysfunction, impaired oxidative metabolism, and elevated ROS production^217^,^218^. Mechanistically, SIRT3 strengthens mitochondrial function by deacetylating key metabolic enzymes such as SOD2 and IDH2, thus optimizing TCA cycle efficiency and preserving cardiomyocyte viability under stress conditions, particularly in hypertensive HF models^219^. Uniquely among class III HDACs, SIRT3 attenuates pathological cardiac remodeling through FOXO3A activation, contrasting with the functional diversity exhibited by other HDAC subtypes in myocardial regulation^220^.

Pharmacological potentiation of SIRT3 *via* NAD^+^-dependent pathways and gut-derived metabolites such as indole-3-propionic acid, has been shown to enhance myocardial bioenergetics and improve diastolic function, especially in HF with preserved ejection fraction^221^-^223^. In preclinical models, SIRT3 overexpression significantly mitigates cardiac hypertrophy and fibrosis, whereas SIRT3 deficiency promotes the development of hypertrophic cardiomyopathy and progressive ventricular dysfunction^224^,^225^.

At the molecular level, SIRT3 orchestrates multiple cardioprotective pathways, including the FOXO3a-dependent transcriptional activation of MnSOD and CAT to counteract ROS accumulation^226^, inhibition of cyclophilin D-dependent mitochondrial permeability transition pore opening to preserve mitochondrial integrity and prevent apoptosis^227^, and interaction with the long noncoding RNA DACH1 to regulate mitochondrial oxidative damage and cell death^228^.

Beyond its roles in mitochondrial function and apoptosis, SIRT3 also suppresses myocardial fibrosis *via* inhibition of p53 acetylation and ferroptosis, along with deacetylation of pro-fibrotic genes^229^-^231^, such as COL1A1 and TGF-β1, ultimately reducing ECM deposition in pressure-overloaded cardiomyocytes^126^. Additionally, SIRT3 contributes to maintaining mitochondrial morphology through optic atrophy 1 (OPA1) deacetylation, maintaining cristae structure and preventing inflammasome activation in cardiac fibroblasts^232^. Through modulation of the β-catenin/peroxisome proliferator-activated receptor (PPAR)-γ and TGFβ/Smad3 axis^233^, SIRT3 further suppresses fibrotic remodeling and supports cardiac function^128^,^234^.

Despite its well-established cardioprotective functions, the temporal dynamics and tissue-specific roles of SIRT3 in different HF phenotypes remain incompletely understood. Future research should investigate strategies for cell-specific delivery of SIRT3 activators to cardiomyocytes and fibroblasts, as well as assess the translational potential of microbiota-derived SIRT3 modulators in human HF. These efforts might offer innovative therapeutic strategies leveraging SIRT3 as a metabolic and epigenetic checkpoint in HF.

### 2.3 SIRT3 and diabetes mellitus

Aging and diabetes converge to induce comparable patterns of multiorgan dysfunction through overlapping molecular pathways^235^, with mitochondrial dysfunction emerging as a central pathogenic mechanism driving the onset and progression of diabetic complications^236^,^237^. As the predominant mitochondrial NAD^+^-dependent deacetylase, SIRT3 serves as a metabolic gatekeeper, and is consistently found to be downregulated in clinical diabetic tissues, where its deficiency correlates with insulin resistance and disturbances in systemic energy homeostasis^238^-^240^.

In skeletal muscle, SIRT3 deficiency impairs insulin-stimulated glucose translocation, aggravating peripheral insulin resistance and contributing to the pathophysiology of type 2 diabetes^241^. Conversely, restoring SIRT3 expression *via* lifestyle interventions or fibroblast growth factor-21 signaling improves mitochondrial integrity and myocardial function^57^, pointing to a promising avenue for metabolic reprogramming in diabetic patients.

Growing evidence supports a cardioprotective role of SIRT3 in diabetic cardiomyopathy (DCM)^242^,^243^, where its activation improves mitochondrial respiratory capacity and suppresses oxidative stress, primarily through modulation of the AGO2/cytochrome b (CYTB) signaling pathway^237^. This regulatory mechanism uncouples excessive glucose levels from ETC dysfunction^237^, whereas SIRT3 downregulation leads to a breakdown of this axis, resulting in impaired ETC activity and accelerated progression of DCM^244^,^245^.

Within the central nervous system, chronic hyperglycemia suppresses SIRT3 expression in the hippocampus, contributing to cognitive deficits through mitochondrial Ca^2+^ overload and neuronal apoptosis^246^. SIRT3 overexpression mitigates neuronal function by inhibiting the VDAC1/GRP75/IP3R complex, thereby reducing mitochondria-associated endoplasmic reticulum membrane formation and protecting neurons from metabolic stress-induced apoptosis^247^.

Under conditions of hyperlipidemia and inflammation, SIRT3 also plays a pivotal role in preserving pancreatic β-cell viability and promoting osteogenic differentiation, thereby exerting antioxidative effects essential for the redox-modulating effects of irisin in diabetic periodontitis models^248^. Lentivirus-mediated SIRT3 silencing abolishes irisin's osteoprotective efficacy against osteoclastic alveolar bone resorption and ROS overproduction, establishing SIRT3 as the obligatory signaling node for irisin-mediated redox homeostasis and bone preservation^240^.

These findings underscore SIRT3's fundamental role in maintaining redox homeostasis and structural integrity across metabolically active organs.

Despite these organ-specific findings, a comprehensive understanding of SIRT3's integrated role in systemic diabetic complications remains incomplete. Future studies should prioritize characterizing SIRT3's cell type-specific SIRT3 functions in diabetic heart, brain, pancreas, and bone tissue, as well as developing targeted therapeutics (*e.g.*, activators, gene therapy) capable of tissue-selective SIRT3 delivery. Ultimately, SIRT3 may represent a convergent therapeutic target for mitigating the multiorgan sequelae of diabetes *via* its dual roles in mitochondrial regulation and oxidative stress control (Fig.3).

### 2.4 SIRT3 and cancer

Aging represents an irreversible carcinogenic risk factor, with epidemiological data revealing an exponential rise in cancer incidence with advancing age^249^.

As a mitochondrial deacetylase, SIRT3 serves as a molecular nexus intersection of cellular senescence and oncogenesis, exerting tumor-suppressive effects by preserving genomic integrity through multiple regulatory mechanisms^250^,^251^. During early oncogenic transformation, SIRT3 stabilizes chromosomal integrity *via* OGG1-mediated DNA repair potentiation through lysine deacetylation^252^,^253^ and orchestrates chromatin remodeling by facilitating H3K56 deacetylation to enhance NHEJ fidelity^55^. Collectively, these mechanisms highlight SIRT3's role as a genomic sentinel in aging tissues prone to malignant transformation.

Beyond its role in genome stability, SIRT3 exerts metabolic control in a context-dependent manner, predominantly acting as a tumor suppressor. For instance, SIRT3 destabilizes hypoxia-inducible factor 1α, thereby attenuating the Warburg effect, a characteristic metabolic adaptation of rapidly proliferating tumors^254^-^256^. Transcriptomic profiling in castration-resistant prostate cancer reveal that SIRT3 suppresses aconitase 2 activation, disrupting glutamine-driven lipogenesis^257^.

In parallel, SIRT3 enhances OXPHOS efficiency *via* ETC assembly optimization in neoplasms, unveiling potential therapeutic vulnerabilities in OXPHOS-dependent malignancies such as pancreatic adenocarcinoma and BRAF-mutated melanomas^258^-^261^. However, emerging evidence indicates that SIRT3 can also exert oncogenic effects in certain genetic or microenvironmental contexts^262^, often mediated by post-translational modifications of metabolic enzymes and stress-response proteins^258^,^263^,^264^. For instance, SIRT3 catalyzes Lys228 deacetylation on pyrroline-5-carboxylate reductase 1, facilitating proline biosynthesis, a critical process for tumor cell proliferation^265^. In colorectal malignancies, SIRT3 optimizes serine metabolism *via* serine hydroxymethyltransferase 2 deacetylation at Lys95, enhances tumor aggressiveness^266^,^267^. Similarly, in glioblastoma models, SIRT3 promotes nucleotide biosynthesis by deacetylating glycine decarboxylase at Lys514, 268,269. SIRT3 also plays a critical role in modulating ROS homeostasis, which serve as a double-edged sword in tumor progression^270^.By deacetylating IDH2 at K143, SIRT3 suppresses ROS-mediated mutagenesis and tumorigenesis^271^.

Conversely, glioma stem cells inactivate SIRT3 to bypass oxidative growth constraints^269^. In chronic lymphocytic leukemia, SIRT3 enables metabolic adaptation confers chemoresistance *via* ROS buffering^272^, whereas in mammary carcinoma, SIRT3 inhibits Src kinase oxidation to suppress metastatic dissemination^125^.Moreover, SIRT3 exerts pleiotropic control over tumor microenvironmental reprogramming and cell death pathways, including fine-tuning apoptotic execution, autophagic quality control, and ferroptotic vulnerability landscapes^273^-^275^. Genetic ablation models confirmed that SIRT3 preserves cellular stress defense *via* activating key unfolded protein response mediators such as FOXO3a and MnSOD, ultimately restraining invasive tumor phenotypes^276^-^278^.

Despite extensive characterization, the dualistic, context-dependent roles of SIRT3 in cancer biology remain incompletely resolved. It has hypothesized that these opposing functions reflect a dynamic interplay between SIRT3's modulation of metabolic plasticity and redox equilibrium, influenced by tumor type, developmental stage, and microenvironmental conditions. Future research should focus on dissecting the spatiotemporal regulation of SIRT3 activity, as well as identifying interacting molecular co-factors or modifications that determine its tumor-suppressive versus pro-tumorigenic roles. Importantly, the development of context-specific SIRT3 modulators may offer a promising strategy for personalized cancer therapies (Fig.3).

### 2.5 SIRT3 and kidney diseases

As metabolically active organs, the kidneys sustain extensive mitochondrial networks to fuel filtration and reabsorption *via* robust energy production^279^. SIRT3 has emerged as a pivotal coordinator of renal bioenergetics, as it maintains mitochondrial homeostasis, enhances antioxidant defenses, and modulates ECM dynamics to counteract oxidative stress and fibrotic remodeling^280^. Clinical and experimental evidence consistently demonstrates that SIRT3 expression is reduced across various nephropathies, and its expression is negatively correlated with histological injury severity^281^. Conversely, SIRT3 overexpression was found to alleviate both acute kidney injury (AKI) and chronic kidney disease (CKD) progression in multiple preclinical models^282^(Fig.3).

#### 2.5.1 SIRT3 and AKI

AKI is clinically defined by the rapid-onset deterioration of glomerular filtration capacity consequent to irreversible cellular necrosis within nephron components, and serves as a pivotal factor in the transition toward CKD^283^,^284^. Among the mechanistic underpinnings of AKI, mitochondrial dysfunction has emerged as a central contributor to its pathogenesis^285^, SIRT3, a key mitochondrial deacetylase, plays a protective role by safeguarding mitochondrial integrity and bioenergetic function^286^.

In preclinical models of sepsis-induced AKI, SIRT3 knockout exacerbates mitochondrial dysfunction in proximal tubules^287^, accompanied by enhanced epithelial cell apoptosis mediated *via* BAX oligomerization, caspase-3 activation, and BCL-2 network disruption^112^,^288^. These observations establish SIRT3 as a critical checkpoint regulator of mitochondrial apoptosis and oxidative stress equilibrium.

Furthermore, SIRT3 deficiency impairs fatty acid β-oxidation, thereby exacerbating parenchymal cell apoptosis and accelerating renal functional deterioration^289^,^290^. Conversely, pharmacological activation of SIRT3 improves OXPHOS efficiency, promoting ATP synthesis while simultaneously limiting ROS accumulation and lipid peroxidation^288^. Transgenic overexpression of SIRT3 confers renoprotective effects *via* multiple molecular pathways, including upregulation of the Nrf2 antioxidant pathway^291^, inhibition of NF-κB signaling through IκBα stabilization^292^, and preservation of tubular epithelial viability^293^. These interventions maintain mitochondrial membrane potential stability while suppressing cytochrome c-mediated apoptotic cascades^294^, ultimately attenuating ischemia-reperfusion (I/R)-induced mitochondrial damage in renal epithelia^295^.

In unilateral ureteral obstruction models, SIRT3 deficiency leads to aberrant acetylation of mitochondrial proteins and increased interstitial collagen accumulation, highlighting its involvement in ECM remodeling^296^. Furthermore, SIRT3 knockout accelerates the transition from AKI to CKD *via* early activation of the TGF-β/Smad3 pathway^297^,^298^, further supporting its role in renal fibrosis initiation and progression. In I/R-induced AKI, targeted SIRT3 activation produces multifaceted nephroprotective effects, including deacetylation of mitochondrial SOD2, enhancing antioxidative capacity, and suppressing pathological superoxide accumulation^299^. Concomitantly, SIRT3 activation also restores mitochondrial ATP synthase complex integrity and cristae morphology, thereby mitigating tubular epithelial apoptosis^300^. Additionally, SIRT3 attenuates inflammatory responses by inhibiting NLRP3 inflammasome activation and downregulating TGF-β1-driven fibrogenesis^293^, further supporting its anti-inflammatory and anti-fibrotic roles.

Intriguingly, metabolomic profiling of SIRT3-deficient specimens from models of AKI reveal elevated levels of glutathione biosynthesis precursors, suggesting a compensatory adaptation to oxidative stress^301^. Moreover, pharmacological AMPK activation using metformin promotes SENP1-mediated deSUMOylation of SIRT3, enhancing its mitochondrial localization and functional activity, which in turn reduces tubular cell apoptosis under metabolic stress^302^.

Collectively, these findings underscore SIRT3 as a master regulator of renal resilience, operating at the intersection of metabolic reprogramming, redox regulation, and anti-fibrotic signaling pathways. Future investigations should aim to unravel the tissue-specific post-translational modifications that fine-tune SIRT3 activity, as well as the potential for SIRT3-targeting agents within the AKI-CKD continuum for precision nephrology.

#### 2.5.2 SIRT3 and CKD

CKD is defined as a progressive renal condition marked by sustained impairment in kidney function for at least 3 months^303^. SIRT3 orchestrates mitochondrial network remodeling, biogenetic processes, and metabolic adaptation to preserve cellular homeostasis, thereby supporting renal repair mechanisms and attenuating fibrotic pathogenesis^304^-^306^. Within the tubular epithelium, SIRT3 suppresses epithelial-mesenchymal transformation (EMT) through the activation of FOXO3a, a transcription factor that directly induces mitochondrial SOD2 and peroxisomal catalase expression^213^. This coordinated antioxidant enzyme induction mitigates intracellular oxidative stress, ultimately preventing the deposition of ECM components associated with early fibrotic lesions^225^,^307^. Moreover, SIRT3 acts as a downstream of UCP1, thereby stabilizing proteins and reducing ROS generation, which further contributes to inhibiting EMT and ECM deposition^213^. The regulatory capacity of SIRT3 extends to fibrogenesis through its ability to disrupt the NF-κB/TGF-β1/Smad axis^139^. Notably, SIRT3 deacetylates β-catenin, a pivotal transcriptional coactivator in fibroblast activation, facilitating the expression of MMP-7^308^ and plasminogen activator inhibitor-1^309^, two mediators implicated in ECM remodeling *via* EMT induction^296^. In the context of angiotensin II-induced nephropathy, SIRT3 exhibits nephroprotective by counteracting iron overload and inhibiting NADPH oxidase-mediated ROS overproduction, ultimately attenuating renal fibrogenesis and delaying CKD progression^310^.

Vascular calcification (VC), a hallmark of advanced CKD^311^, is another pathological process in which SIRT3 exerts protective roles. SIRT3 counteracts VC *via* mitochondrial function preservation and oxidative stress mitigation^312^. Notably, SIRT3 deacetylates proteins within the PGC-1α/TFAM pathway, promoting mitochondrial biogenesis while attenuating vascular smooth muscle cell calcification^313^. Genetic ablation studies demonstrated that the abrogation of VC is completely abolished in SIRT3-deficient models, whereas AMPK/SIRT3 axis activation attenuated mineralization through mitochondrial functional restoration and oxidative stress mitigation^314^. Biochemical analyses delineated a regulatory axis in which soluble epoxide hydrolase (sEH) modulates SIRT3 turnover *via* proteolytic degradation, with sEH deficiency preserving SIRT3 bioavailability to fulfill bioenergetic demands and impede renal VC progression^315^.

Taken together, these findings highlight SIRT3 as a master regulator of renal and vascular resilience in CKD. Enhancing SIRT3 activity may provide a promising therapeutic strategy to counteract mitochondrial dysfunction, fibrosis, and VC in affected patients. Future research should focus on developing selective SIRT3 activators, exploring cell-specific functional roles, and deciphering how post-translational modifications affect SIRT3's stability, localization, and bioactivity.

### 2.6 SIRT3 and age-related hearing loss (AHL)

AHL, the most prevalent form of auditory impairment among older adults, is tightly correlated with cumulative oxidative stress^349^. CR, a well-established anti-aging intervention, has been shown to alleviate AHL by reducing oxidative damage in cochlear cells^350^. However, this protective effect is abolished in SIRT3-deficient mice^351^, highlighting the indispensable role of SIRT3 in mediating CR-induced auditory resilience. Mechanistically, SIRT3 enhances the activity of IDH2 under CR, thereby boosting mitochondrial NADPH production and facilitating efficient ROS clearance^352^. This mitochondrial antioxidant defense mechanism plays a crucial role in preserving cochlear hair cell integrity and delays the progression of hearing loss^353^.

Overall, these findings underscore SIRT3 as a critical mediator of mitochondrial redox homeostasis within the auditory system. In light of the absence of effective interventions for AHL, SIRT3 represents a promising therapeutic target for aging-related hearing disorders. Future research should explore whether pharmacological activation of SIRT3 can replicate the benefits of CR in the cochlea, as well as explore its potential as a biomarker to predict AHL susceptibility (Fig.3).

### 2.7 SIRT3 and chronic obstructive pulmonary disease (COPD)

COPD is a progressive and largely irreversible respiratory disorder, representing a leading global cause of morbidity and mortality^316^. Aging has been identified as a key contributor to the pathogenesis of COPD^317^,^318^, primarily through its effects on oxidative stress regulation^319^ and mitochondrial dysfunction^320^.

SIRT3 is critical in maintaining mitochondrial homeostasis and counteracting oxidative stress, both of which are critical in the onset and progression of COPD^321^. The PGC-1α/SIRT3 signaling pathway is especially pivotal for mitochondrial biogenesis and redox equilibrium^322^. In murine models, SIRT3 deficiency exacerbates alveolar destruction, increases airway inflammation, and accelerates the declines in pulmonary function^48^, underscoring its protective effects in respiratory health. Mechanistically, SIRT3 enhances MnSOD activity through lysine deacetylation, thereby attenuating oxidative damage within mitochondria of airway epithelial cells^323^.

In cigarette smoke (CS)-induced lung injury models, SIRT3 upregulation promotes the deacetylation of SOD2, which reduces oxidative stress and attenuates lung structural damage and functional deterioration^324^. Conversely, SIRT3 inhibition modulates the Nrf2 pathway, decreasing inducible nitric oxide synthase expression and ROS accumulation, while activation of SIRT3 provides protection against CS-induced ferroptosis in bronchial epithelial cells^325^.Moreover, the expression of SIRT3 is regulated by microRNA pathways, which are closely linked to cellular senescence. Under oxidative stress conditions, miR-494-3p directly targets SIRT3 in small airway epithelial cells, leading to downregulation of SIRT3 levels, increased expression of p27, and accelerated cellular senescence, all of which contribute to the pathophysiology of COPD 49.

**Figure 4:**
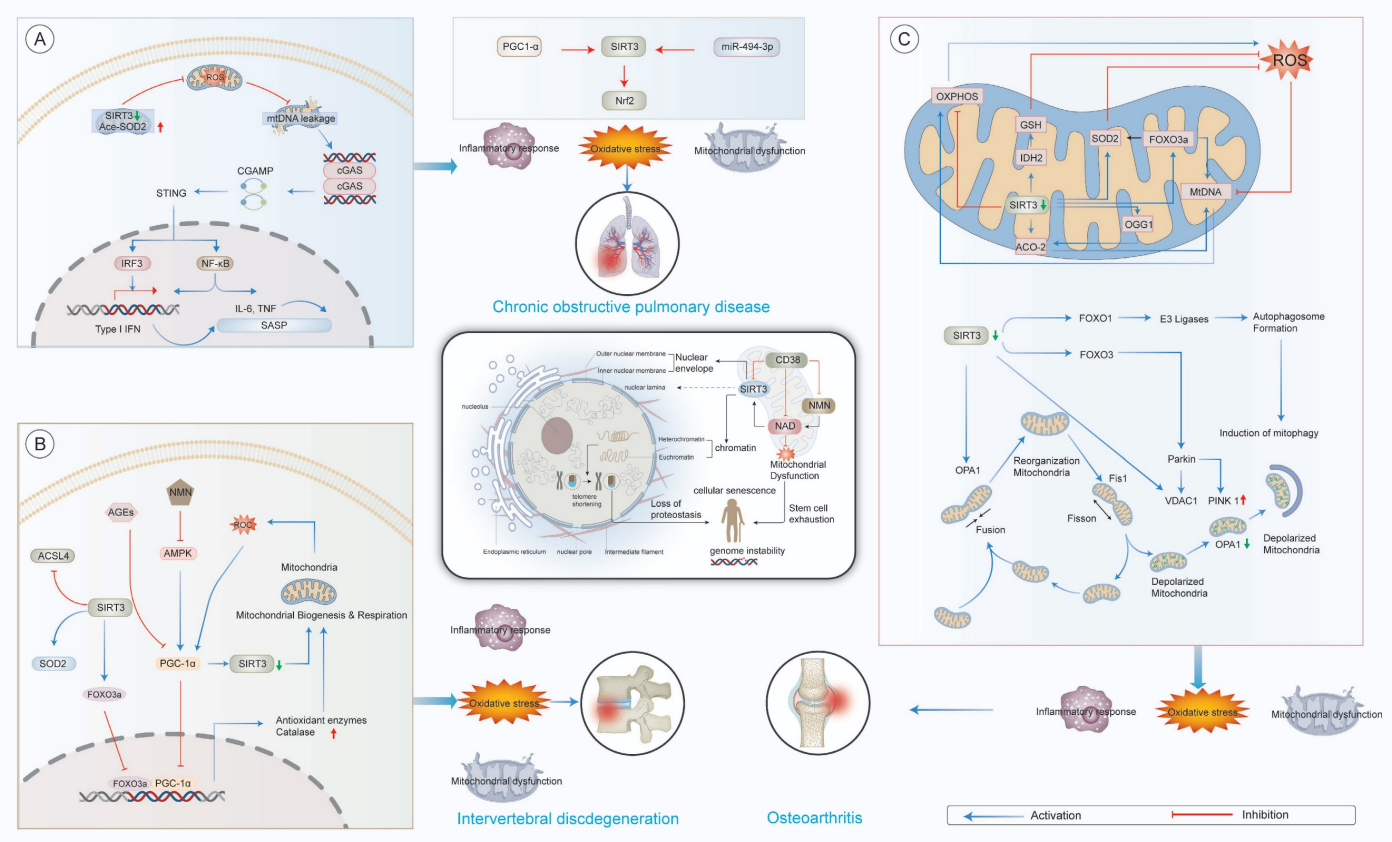
** Modulatory mechanisms of SIRT3 in aging-related pathologies. (A) Chronic Obstructive Pulmonary Disease (COPD).** Mitochondrial dysfunction induces excessive ROS production and mtDNA leakage, which activates the cGAS-STING pathway and triggers inflammatory cytokine release (e.g., IL-6, TNF). This promotes cellular senescence and the senescence-associated secretory phenotype (SASP). Impaired SIRT3 activity further aggravates oxidative stress by reducing SOD2 deacetylation, weakening mitochondrial antioxidant defenses and amplifying inflammation. **(B) Intervertebral Disc Degeneration (IDD).** Metabolic stress and AGE accumulation disrupt mitochondrial biogenesis and respiration. The AMPK-SIRT3-PGC-1α axis is central to maintaining mitochondrial function and antioxidant capacity. Its dysregulation leads to reduced expression of enzymes like SOD2 and catalase, increased ROS levels, and nucleus pulposus cell senescence, contributing to disc degeneration. **(C) Osteoarthritis (OA).** Oxidative stress impairs mitochondrial dynamics and activates the PINK1/Parkin-dependent mitophagy pathway. Inadequate mitophagy results in accumulation of dysfunctional mitochondria, elevated ROS, and chondrocyte apoptosis. Disrupted mitochondrial quality control exacerbates inflammation and cartilage matrix degradation, accelerating joint degeneration.

In conclusion, these findings emphasize the multifaceted role of SIRT3 in COPD, primarily through its functions in preserving mitochondrial integrity, suppressing oxidative injury, and delaying senescence in airway epithelial cells. These mechanisms are particularly relevant in the context of aging lung and environmental insults like CS. Future research should focus on developing therapeutic approaches that target SIRT3 activation or its downstream signaling pathways and further explore its interactions with non-coding RNAs and ferroptosis-related processes, particularly within the context of aging and environmental exposures. Such investigations will improve the understanding of SIRT3's contribution to pulmonary resilience, and pave the way for precision therapies aimed at treating aging-related COPD(Fig.4).

### 2.8 SIRT3 and degenerative spine and joint diseases

The musculoskeletal system plays a critical role in providing structural integrity and enabling movement^326^. Aging-related disruptions in bone homeostasis are commonly associated with an increased risk of degenerative conditions, such as intervertebral disc degeneration (IDD) and osteoarthritis (OA)^327^. These disorders are primary contributors to chronic pain and disability in older populations, significantly diminishing quality of life and imposing considerable socioeconomic burdens^328^.

Consequently, identifying molecular regulators, such as SIRT3, which may serve as potential therapeutic targets, is of paramount importance (Fig.4).

#### 2.8.1 SIRT3 and IDD

Cellular senescence, mitochondrial dysfunction, and oxidative stress are key mechanisms involved in the pathogenesis of IDD^329^. SIRT3 expression is significantly reduced in degenerated intervertebral discs, and its knockdown further exacerbates the deterioration of nucleus pulposus cells (NPCs) under oxidative stress conditions^330^.Emerging studies suggest that lactate can downregulate SIRT3, which, in turn, increases the acyl-CoA synthetase long chain family member 4 lactylation, thereby promoting ferroptosis and accelerating the decline of NPC functionality^331^. Moreover, SIRT3 deficiency amplifies ROS-induced damage^332^, whereas SIRT3 overexpression has been shown to alleviate IDD severity *in vivo*^20^,^330^,^333^, highlighting its protective role.

Therapeutic strategies, such as hydrogel microspheres designed to upregulate both SIRT3 and SOD2, have exhibited promise in maintaining NPC viability^334^. Additionally, SIRT3 safeguards against advanced glycation end product-induced apoptosis^335^ and supports mitochondrial homeostasis *via* the AMPK/PGC-1α pathway^336^.

Taken together, these findings indicate that SIRT3 serves as a crucial regulator of NPC survival, orchestrating mitochondrial quality control, redox homeostasis, and apoptotic pathways. Despite these advances, the potential of SIRT3 as a biomarker for monitoring IDD progression or as a direct therapeutic target remains uncertain. Future studies should explore its role in human clinical samples, examine tissue-specific effects, and investigate the efficacy of SIRT3-activating compounds in translational models.

#### 2.8.2 SIRT3 and OA

Mitochondrial dysfunction is increasingly recognized as a defining characteristic of OA^337^. SIRT3 preserves mitochondrial integrity by deacetylating cytochrome c oxidase subunit 4 isoform 2^338^, with its deletion correlating with the exacerbation of OA in both animal and human models^339^,^340^.

As aging progresses, autophagic activity declines, contributing to chondrocyte loss^341^. SIRT3 promotes mitophagy *via* the PINK/Parkin axis and maintains mitochondrial health by regulating FOXO3a hyperacetylation^342^. Furthermore, SIRT3 inhibits chondrocyte apoptosis through the PI3K/Akt/mTOR signaling pathway^147^.

Oxidative stress is another central pathological factor in OA^343^. SIRT3 facilitates rapid ROS clearance through antioxidant protein deacetylation and long-term redox homeostasis *via* FOXO3a activation^344^. Notably, reduced SIRT3 activity leads to SOD2 hyperacetylation, which results in increased ROS accumulation^345^.

Recent studies indicate that epigenetic modifications impair SIRT3 function^346^, such as through SUMOylation-mediated inhibition, thereby exacerbating mitochondrial dysfunction and cartilage degeneration^347^. SIRT3 also promotes mitochondrial fusion, facilitating mtDNA complementation and enhancing cartilage resilience^348^.

In summary, SIRT3 plays a multifaceted role in OA by regulating mitochondrial biogenesis, redox signaling, and chondrocyte viability. Nevertheless, the potential of SIRT3 activation as a disease-modifying strategy remains unclear. Future research should focus on elucidating the upstream regulatory mechanisms of SIRT3 in aging cartilage and evaluating the therapeutic potential of SIRT3-targeting interventions in both preclinical and clinical trials.

## 3. SIRT3 and therapeutic targets

The aforementioned evidence delineated the multifaceted regulatory roles of SIRT3 in biological aging processes and associated geriatric comorbidities. Its therapeutic potential is particularly evident in the context of neurodegenerative diseases, CVD, diabetes mellitus, and cancer, as well as kidney diseases. Accumulating research has focused on the pharmacological targeting of SIRT3, with the development of activators aimed at addressing these pathophysiological conditions that impose significant clinical burdens (Table 1).

### 3.1 Natural modulators

#### 3.1.1 HKL

HKL, a naturally occurring bisphenolic lignan isolated from *Magnolia officinalis*, is a potent SIRT3 activator^354^. Previous studies revealed that HKL upregulates SIRT3 while augmenting its enzymatic activity^355^. Preclinical studies demonstrated that HKL can ameliorate pre-existing cardiac hypertrophy and suppresses SIRT3-dependent cardiac fibroblast proliferation^218^, suggesting that the beneficial effects of HKL are SIRT3-dependent.

Mechanistically, HKL-induced activation of SIRT3 mitigates silica-induced fibrosis and mtDNA damage *via* the cGAS/STING pathway^356^, providing further evidence for its therapeutic potential in conditions involving cellular damage and inflammation. Additionally, HKL possesses neuroprotective properties, likely attributable to its ability to cross the blood-brain barrier as a small molecule^357^,^358^. A prior study indicated that HKL promotes mitochondrial fusion and supports neural survival *via* the SIRT3/AMPK pathway in models of subarachnoid hemorrhage^359^. Moreover, HKL protects the brain from I/R injury in mice by reducing ROS production and enhancing mitochondrial function^360^. Furthermore, HKL ameliorates intracerebral hemorrhage-induced apoptosis and mitochondrial fission *via* SIRT3 activation^354^.

In murine models, HKL-activated SIRT3 upregulates mitochondrial GPX4 and decreases its acetylation, thereby inhibiting neuronal ferroptosis and mitigating perioperative neurocognitive disorders following anesthesia and surgery^361^. These findings further support the critical role of SIRT3 in regulating pyruvate dehydrogenase E1α deacetylation, bridging glycolysis and the TCA cycle in tubular epithelial cells during the progression of renal fibrosis^362^.

In conclusion, these findings highlight the promising therapeutic potential of HKL as a novel SIRT3-targeted agent for the prevention or even reversal of cardiac, pulmonary, and neurodegenerative diseases. However, further clinical investigations are required to evaluate its safety, efficacy, and broader applications in human diseases.

#### 3.1.2 Resveratrol (RSV)

RSV, a phytoalexin extracted from *Veratrum grandiflorum*, has been reported to broadly activate SIRT proteins^363^. RSV significantly diminishes mtROS generation by enhancing the accumulation of SIRT3 within mitochondria, which subsequently upregulates of FOXO3A-dependent transcription of mitochondrial genes, including ATP6, CO1, Cytb, ND2, and ND5, which in turn improves complex I activity and ATP synthesis^364^.

In addition, RSV increases the expression of phosphorylated AMPK, PGC-1α, and SIRT3, as well as enhance SIRT3 transcription through the estrogen-related receptor-α (ERRα)-dependent transcription^365^. The beneficial of RSV on mitochondrial redox balance were abrogated when cells were treated with an AMPK inhibitor or transfected with siRNA targeting AMPK, PGC-1α, or SIRT3,suggesting that these protective mechanisms are mediated through the AMPK/PGC/1α/ERRα/SIRT3 signaling pathway^366^. This cascade ultimately helps attenuate oxidative injury in endothelial cells. In myocardial I/R injury models, RSV activates the SIRT3/FoxO pathway and downstream factors, such as Mfn2, Parkin, and PGC-1α, which together contribute to the restoration of mitochondrial integrity and normalization of autophagic flux^367^. Additionally, RSV alleviates cadmium-induced ultrastructural abnormalities and mitochondria dysfunction by upregulating SIRT3 expression, reversing the repression of PGC-1α, Nrf1, and TFAM, and PINK1/Parkin-mediated mitophagy initiation^31^. Experimental investigations have also elucidated the therapeutic efficacy of RSV in attenuating sepsis-induced AKI an a SIRT3/SOD2-dependent manner^368^, thereby maintaining mitochondrial homeostasis. Mechanistic studies further described the RSV-mediated modulation of SIRT3/FOXO3a signaling, leading to the transcriptional activation of PGC-1α and SOD2^369^,^370^.

#### 3.1.3 Other natural compounds

Curcumin modulates SIRT3 activity by coordinating mitochondrial quality control mechanisms, including bioenergetic regulation, optimization of mitochondrial dynamics, selective autophagy, and redox equilibrium^371^. These multifaceted actions highlight its therapeutic potential in preserving mitochondrial function and mitigating aging-related diseases. The Asteraceae-derived flavonoid silybin can attenuate cisplatin-induced nephrotoxicity by preserving renal tubular integrity and mitochondrial bioenergetics, likely *via* SIRT3-mediated mechanisms^372^, highlighting the potential therapeutic candidate of silybin in kidney-related pathologies. The *Sophora flavescens*-derived alkaloid matrine engages its protective effects against cisplatin-induced AKI *via* modulating mitochondrial membrane dynamics through the SIRT3/OPA1 pathway, combining antioxidant and anti-inflammatory mechanisms to preserve renal function^373^. Beyond nephroprotection, matrine also confers cardiovascular benefits *via* enhancing SIRT3/AMPK pathway, which preserving cardiomyocyte viability and limiting cell death in murine models^374^.

The licorice-derived compound liquiritigenin induces Nrf2 translocation, thereby potentiating SIRT3 activity and facilitating mitochondrial biogenesis while simultaneously suppressing apoptosis in AKI models^375^. This dual action highlights liquiritigenin's therapeutic value in mitigating oxidative stress injury and cell death. Catechins stimulate the SIRT3/SOD2 pathway through PPAR-α activation while stimulating ketogenesis-SIRT3 crosstalk concurrently to mitigate oxidative insults in diabetic nephropathy^376^. However, contrasting findings indicate that in cancer cells, catechins may repress SIRT3 expression at both mRNA and protein levels through ERRα modulation^377^, suggesting their biological effects are highly context-dependent. The dietary flavonoid apigenin, ubiquitous in plant-based diets, exerts nephroprotective effects by elevating the intracellular NAD^+^/NADH ratio and promoting SIRT3-mediated CD38 downregulation in diabetic kidney disease models^378^. These findings underscore apigenin's therapeutic potential as a metabolic modulator in nephropathy. Finally, *Poria cocos*-derived poricoic acid A upregulates SIRT3 and promotes β-catenin K49 deacetylation, effectively attenuating renal fibroblast activation and renal interstitial fibrosis *in vivo* and *in vitro*^296^, suggesting poricoic acid A may serve as a promising therapeutic candidate in fibrosis-related renal diseases.

### 3.2 Bioactive substances

#### 3.2.1 2-Acetylphenolquinone congener (2-APQC)

Fu *et al.* identified 2-APQC as a novel pharmacological agonist of SIRT3. In a series of integrated preclinical models spanning cellular and organismal levels, 2-APQC significantly alleviated isoproterenol-induced pathological hypertrophy and fibrotic progression, whereas its efficacy was completely lost in SIRT3-knockout models, establishing causal dependence. Additionally, SIRT3 inhibition *via* NAM markedly attenuated the anti-hypertrophic effects of 2-APQC, thereby confirming SIRT3 as its primary molecular target. Mechanistically, further investigations delineated the multifaceted regulatory effects of SIRT3 activation, which worked in tandem with the suppression of key signaling pathways, including AKT/mTOR/p70S6K and JNK/TGF-β/Smad3, to attenuate pathological cardiac remodeling. This approach specifically targeted hypertrophic responses and maladaptive fibrotic deposition, suggesting a potential therapeutic avenue for targeted cardiac intervention^379^.

#### 3.2.2 Melatonin

Accumulating evidence indicates that melatonin upregulates SIRT3, which in turn promotes mitophagy to mitigate oxidative stress-induced damage^380^. In preclinical models, exogenous melatonin supplementation preserved mitochondrial integrity and activate autophagy, thereby attenuating sepsis-induced multiorgan dysfunction through SIRT3 upregulation^381^. Notably, SIRT3 knockout models completely abolished the therapeutic benefits of melatonin against contrast-induced AKI, highlighting the essential role of SIRT3 in mediating melatonin's protective actions^382^.

Moreover, melatonin was found to effectively counteract adverse left ventricle remodeling and mitigate cardiac dysfunction in DCM. These benefits are attributed to the promotion of autophagy, reduction of apoptosis, and mitigation of mitochondrial dysfunction, which facilitated by the inhibition of Mst1 phosphorylation and upregulation of SIRT3 expression in DCM model^383^. Mechanistically, melatonin specifically activates the 28-kDa isoform of SIRT3, which enhances its enzymatic activity within mitochondria, thereby boosting its intramitochondrial catalytic efficiency^384^.

In depth analyses have outlined melatonin's orchestration of sequential mitophagy processes, with the SIRT3-mediated deacetylation of TFAM at Lys154 acting as the pivotal molecular switch to promote mitophagic flux^382^. Furthermore, endogenous melatonin demonstrates notable cytoprotective capacity through its involvement in the redox-sensitive SIRT3/FOXO3α/ROS regulatory triad, thereby effectively reducing macrophage cytotoxicity under metabolic-stress conditions^385^. Additionally, melatonin induced SOD2 deacetylation, leading to a reduction in oxidative stress, a process that was completely blocked in the absence of SIRT3^381^. The AMPK/PGC1α/SIRT3 axis has also been identified as a pivotal pathway in melatonin's protective effects against myocardial IR injury in rats with type 1 diabetes, primarily by preserving mitochondrial function^386^. Collectively, these findings establish melatonin's SIRT3-dependent therapeutic efficacy, underscoring its potential for clinical translation in treating diseases associated with mitochondrial dysfunction and oxidative stress.

#### 3.2.3 Other bioactive compounds

Zhang *et al.* designed and synthesized an innovative class of pseudo-natural macrocyclic sulfonamides derivatives. In murine PD models, spiro-grafted macrocyclic sulfonamide 2a treatment significantly attenuated spontaneous locomotor deficits and motor coordination impairment compared to control mice^387^. These findings highlight the potential therapeutic role of SIRT3-dependent mechanisms, suggesting that SIRT3 activation could be pivotal in modulating PD pathologies.

Stanniocalcin-1, a glycoprotein hormone, has been demonstrated to orchestrate a hormetic AMPK/SIRT3 axis that mitigates oxidative stress and apoptosis^388^. This hormetic axis is crucial for maintaining cellular homeostasis and ameliorating renal injury in diabetic murine models by inhibiting the expression of Bnip3 through the AMPK/SIRT3 pathway^389^, further underscoring its therapeutic potential in kidney disease. Similarly, intermedin enhances mitochondrial bioenergetic capacity *via* AMPK/SIRT3-mediated cristae remodeling in VC models^314^, positioning it as a potent therapeutic strategy. Mechanistic investigations revealed that meteorin-like protein activates the PGC-1α/SIRT3 axis, which stimulates AMPK at Thr172 and increases UCP1-dependent proton leak, thereby preserving mitochondrial ultrastructural integrity and regulating energy metabolism, particularly in diabetic nephropathy^207^. Furthermore, the annexin A1-derived tripeptide Ac2-26 was reported to transcriptionally upregulate SIRT3, concurrently reducing oxidative damage while promoting mitochondrial biogenesis and mitophagy^390^.

These findings suggest that SIRT3 activation could be leveraged to enhance mitochondrial function, providing a promising avenue for therapeutic interventions aimed at combating oxidative stress and mitochondrial dysfunction.

## Conclusion and perspective

The available research delineates SIRT3 as a pleiotropic regulatory hub within the cellular senescence and geropathological trajectories, establishing it as a critical determinant in a range of disorders spanning CVDs, neurodegenerative conditions, metabolic syndrome, cancer, kidney diseases, COPD, and degenerative spine and joint diseases. The multifaced regulation of metabolic adaptation, oxidative stress homeostasis, epigenetic integrity, and inflammatory responses by SIRT3 enables it to integrate systemic stress responses, thereby maintaining physiological resilience throughout the aging process.

Emerging evidence underscores SIRT3's pivotal role in preserving mitochondrial function by suppressing excessive ROS generation, maintaining ATP synthesis, and stabilizing mitochondrial dynamics. Additionally, SIRT3's regulation of the mitochondrial acetylome has a profound effect on chromatin remodeling and genome stability, establishing it as a crucial epigenetic modulator. Through these mechanisms, SIRT3 both protects cellular integrity and maintains organ function during aging, making it a promising target for therapeutic intervention.

Pharmacologic potentiation of SIRT3 deacetylase activity represents a burgeoning frontier in translational geroscience. Targeted activation strategies have increased therapeutic viability across multisystem aging-related morbidities. Beyond CR and pan-SIRT activators, innovative small molecules including 2-APQC, melatonin, and pseudo-natural macrocyclic sulfonamides displayed organ-specific protective effects in preclinical models. For instance, these compounds provide cardioprotection through the AMPK/PGC1α axis, neuroprotection *via* SOD2-mediated ROS suppression and renal protection by enhancing mitophagy. These findings both highlight the translational promise of SIRT3 agonism and emphasize its potential in combating mitochondrial dysfunction and oxidative damage, which are key drivers of aging and associated diseases.

Despite these encouraging advances, there remains a significant gap in translating these preclinical successes into clinical applications. The clinical development of SIRT3-targeted interventions remains in its nascent stage, being constrained by the paucity of robust, randomized human studies and the absence of aging-specific clinical endpoints. Priority research directions should adopt a three-pronged approach to bridge this gap, employing adaptive clinical trial designs that incorporate composite geriatric outcomes and functional endpoints relevant to older populations; implementing omics-based biomarker discovery platforms to stratify patients, monitor SIRT3 activity, and validate mechanisms of action; and establishing international longitudinal registries for long-term safety and efficacy surveillance. These methodologically rigorous initiatives will bridge critical knowledge gaps between preclinical findings to clinical implementation in aging populations.

Furthermore, the context-dependent nature of SIRT3 functions warrants further investigation. Although SIRT3 exerts cytoprotective effects in various cellular contexts, its oncogenic potential in specific tissue types and cellular environments must be explored in greater depth. Elucidating its interactions with other mitochondrial regulators and signaling pathways might uncover synergistic therapeutic opportunities. Moreover, the application of single-cell multiomics technologies offers an exciting avenue to dissect the cellular heterogeneity of SIRT3 activities across different tissue types and disease stages. This will provide deeper insights into its roles within both normal aging and pathological contexts, including cancer, neurodegeneration, and CVDs.

In conclusion, SIRT3 represents a compelling target for delaying or even reversing aging and aging-related diseases. By restoring mitochondrial function and mitigating oxidative damage, SIRT3 holds the potential to significantly extend healthspan. Ongoing preclinical and early-phase clinical studies will further support the selective activation of SIRT3 as a therapeutic strategy to improve health outcomes in aging populations. Ultimately, SIRT3 represents a focal point for future geroscience research, offering a promising pathway toward enhancing the quality of life in older adults and mitigating the burden of aging-related conditions.

## Figures and Tables

**Table 1:**
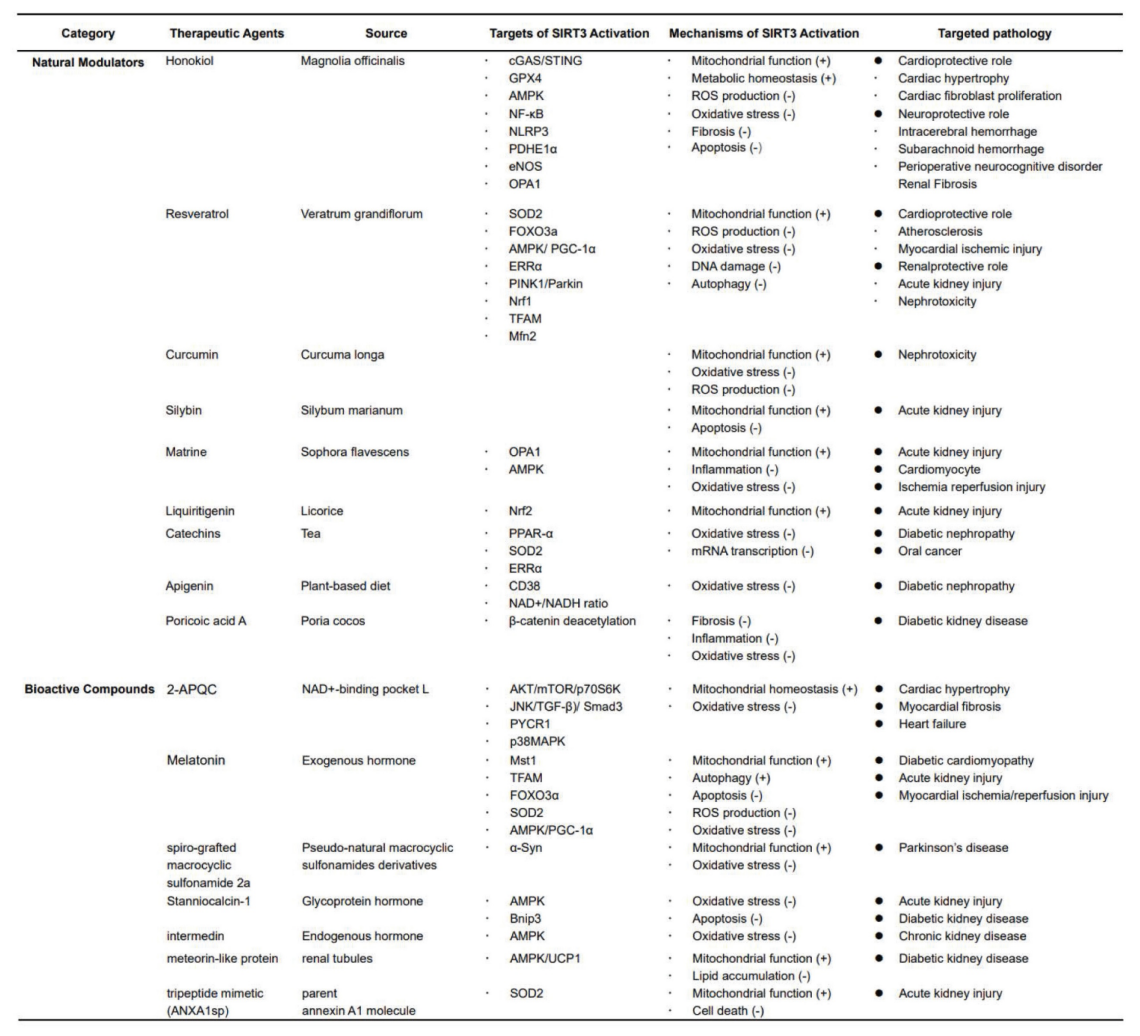
Therapeutic agents targeting SIRT3 activation and their mechanisms of action in various diseases.
